# Complete Genome Assemblies for Three Variants of the *Wolbachia* Endosymbiont of Drosophila melanogaster

**DOI:** 10.1128/MRA.00956-19

**Published:** 2019-11-07

**Authors:** Preston J. Basting, Casey M. Bergman

**Affiliations:** a Institute of Bioinformatics, University of Georgia, Athens, Georgia, USA; b Department of Genetics, University of Georgia, Athens, Georgia, USA; University of Maryland School of Medicine

## Abstract

Here, we report genome assemblies for three strains of Wolbachia pipientis, assembled from unenriched, unfiltered long-read shotgun sequencing data of geographically distinct strains of Drosophila melanogaster. Our simple methodology can be applied to long-read data sets of other *Wolbachia*-infected species with limited *Wolbachia*-host lateral gene transfers to produce complete assemblies for this important model symbiont.

## ANNOUNCEMENT

Wolbachia pipientis is a widespread bacterial endosymbiont that infects 40% of arthropod species (1) and induces a wide range of effects including cytoplasmic incompatibility, feminization, male killing, and parthenogenesis (2). Currently, our understanding of the impact of *Wolbachia* on its hosts is limited by the lack of complete reference genomes for different *Wolbachia* strains, with only 18 of 84 *Wolbachia* assemblies in the NCBI assembly database as of August 2019 defined as complete.

Recently, Faddeeva-Vakhrusheva et al. (3) showed that a complete assembly of *Wolbachia* could be generated as a by-product of assembling the genome of a *Wolbachia*-infected arthropod species using PacBio long-read sequences. Based on this observation, we attempted to generate complete Wolbachia assemblies using long-read shotgun sequencing data for three geographically distinct Drosophila melanogaster lines (I23 from Ithaca, NY; N25 from the Netherlands; and ZH26 from Zimbabwe) (4) that were previously identified by Early and Clark (5) as being infected with variants of the *Wolbachia* strain *w*Mel. These flies were reared on a diet of 10% yeast, 10% glucose, and 1% agar at 25°C (J. Chaston, personal communication). As described by Long et al. (4), DNA was extracted by grinding ∼200 adult flies in liquid nitrogen, transferring to a solution of buffer G2 with 38 μl of RNase A (100 mg/ml) and 500 μl of proteinase K (catalog number 158920; Qiagen), incubating the solution overnight at 50°C, and then extracting DNA using the Qiagen Genomic-tip kit (catalog number 10243). DNA was then sequenced on a PacBio Sequel instrument (Pacific Biosciences, Inc.) using 2 or 3 single-molecule real-time (SMRT) cells per sample. Additionally, public short-read Illumina sequencing was used for the same D. melanogaster lines (5). As described by Early and Clark (5), DNA for these samples was extracted from 50 adult female flies using the Qiagen DNeasy blood and tissue kit. Sequencing was performed on an Illumina HiSeq 2000 instrument to produce 100-bp paired-end reads with a 450- to 500-bp insert size. No quality control steps were applied to the PacBio or Illumina sequencing reads prior to assembly and polishing.

All reads from PacBio whole-genome shotgun sequences of each strain were assembled using CANU v1.8 (genomeSize=137.7m, useGrid=False) (6). Assemblies for each strain generated only one contig matching the *w*Mel reference genome (GenBank accession number NC_002978) by BLASTN v2.9.0 search with default parameters (7), which in each case corresponded to the entire *Wolbachia* genome. Repetitive regions from the ends of uncircularized *Wolbachia* contigs were trimmed using minimus2 from AMOS v3.1.0 with default parameters (8). The trimmed *Wolbachia* contigs were then adjusted using BLASTN v2.9.0 (7) and faFrag (9) with default parameters so that the start of each contig matched the *w*Mel reference start. The contigs were then polished using Arrow (SMRTlink v6.0.0.47841; Pacific Biosciences) and Pilon v1.23 (10) using Illumina reads from Early and Clark (5) (Table 1) mapped to the contigs using BWA-MEM v0.7.17 with default parameters (11).

**TABLE 1:** Accession numbers and statistics for assemblies produced and raw sequencing data used in this study

Assembly	Assembly accession no.	Genome size (bp)	GC content (%)	PacBio read accession no.	No. of PacBio reads^*a*^	Avg PacBio read length (bp)^*a*^	Illumina read accession no.	No. of Illumina reads^*a*^	Avg Illumina read length (bp)^*a*^
I23	CP042444	1,269,137	35.24	SRR7060336	31,867	4,140	SRR1663552	547,585	99
N25	CP042446	1,267,781	35.23	SRR7060337	47,126	4,096	SRR1663577	358,462	98
ZH26	CP042445	1,267,436	35.23	SRR7060339	47,960	4,407	SRR1663599	364,241	98

a Statistics are for the subset of reads from the *Wolbachia* endosymbiont only, not the complete shotgun sequence data set.

After polishing, we identified 54, 13, and 18 single-nucleotide polymorphism (SNP)/indel variants for the I23, N25, and ZH26 *Wolbachia* strains, respectively, relative to the *w*Mel reference genome. The higher similarity of the N25 and ZH26 *Wolbachia* strains and increased divergence of the I23 *Wolbachia* strain relative to the *w*Mel reference genome are consistent with previous work showing that *Wolbachia* genomes from lines N25 and ZH26 are both in clade III of the *w*Mel phylogeny (which also contains the *w*Mel reference genome), while the *Wolbachia* genome from line I23 is in clade I of the *w*Mel phylogeny (which is more divergent from the *w*Mel reference genome) (5, 12).

Our work extends that of Faddeeva-Vakhrusheva et al. (3) by showing that high-quality, complete genome assemblies of *Wolbachia* strains can be generated without experimental enrichment of symbiont DNA (e.g., references 13 and 14). Successful *de novo* assembly of complete *Wolbachia* genomes directly from unenriched long-read sequences also demonstrates that it is unnecessary to computationally filter symbiont reads from host reads based on similarity to *Wolbachia* reference genomes prior to assembly (15, 16). We expect this process to be particularly useful for *Wolbachia*-infected hosts with few host-symbiont lateral gene transfer events, such as D. melanogaster (17), in which there will be few hybrid reads between host and symbiont to confound the assembly process. As the cost of long-read sequencing decreases, we argue that direct sequencing and assembly of unenriched, unfiltered long-read data sets could be applied easily to other *Wolbachia*-infected arthropod and nematode species to expand the number of complete *Wolbachia* reference genomes.

### Data availability.

The assemblies produced in this study were deposited at NCBI under accession number PRJNA557362. PacBio data used to generate these assemblies were published by Long et al. (4) and are available under SRA accession number SRP142531. Illumina data used to polish the assemblies were published by Early and Clark (5) and are available under SRA accession SRP050151. Accession numbers for assemblies produced and raw read data used in this study are given in Table 1.
